# Human metapneumovirus prevalence and patterns of subgroup persistence identified through surveillance of pediatric pneumonia hospital admissions in coastal Kenya, 2007–2016

**DOI:** 10.1186/s12879-019-4381-9

**Published:** 2019-08-30

**Authors:** John W. Oketch, Everlyn Kamau, Grieven P. Otieno, James R. Otieno, Charles N. Agoti, D. James Nokes

**Affiliations:** 1 Kenya Medical Research Institute (KEMRI) -Wellcome Trust Research Programme, Kilifi, KEMRI Centre for Geographic Medicine Research – Coast, Kilifi, Kenya; 2 School of Health and Human Sciences, Pwani University, Kilifi, Kenya; 3 School of Life Sciences, and Zeeman Institute for Systems Biology and Infectious Disease Epidemiology Research (SBIDER), University of Warwick, Coventry, UK

**Keywords:** Human metapneumovirus, Prevalence, Subgroup, Epidemic, Temporal

## Abstract

**Background:**

Human metapneumovirus (HMPV) is an important respiratory pathogen that causes seasonal epidemics of acute respiratory illness and contributes significantly to childhood pneumonia. Current knowledge and understanding on its patterns of spread, prevalence and persistence in communities in low resource settings is limited.

**Methods:**

We present findings of a molecular-epidemiological analysis of nasal samples from children < 5 years of age admitted with syndromic pneumonia between 2007 and 2016 to Kilifi County Hospital, coastal Kenya. HMPV infection was detected using real-time RT-PCR and positives sequenced in the fusion (F) and attachment (G) genes followed by phylogenetic analysis. The association between disease severity and HMPV subgroup was assessed using Fisher’s exact test.

**Results:**

Over 10 years, 274/6756 (4.1%) samples screened were HMPV positive. Annual prevalence fluctuated between years ranging 1.2 to 8.7% and lowest in the recent years (2014–2016). HMPV detections were most frequent between October of one year to April of the following year. Genotyping was successful for 205/274 (74.8%) positives revealing clades A2b (41.0%) and A2c (10.7%), and subgroups B1 (23.4%) and B2 (24.9%). The dominance patterns were: clade A2b between 2007 and 11, subgroup B1 between 2012 and 14, and clade A2c in more recent epidemics. Subgroup B2 viruses were present in all the years. Temporal phylogenetic clustering within the subgroups for both local and global sequence data was seen. Subgroups occurring in each epidemic season were comprised of multiple variants. Pneumonia severity did not vary by subgroup (*p = 0.264*). In both the F and G gene, the sequenced regions were found to be predominantly under purifying selection.

**Conclusion:**

Subgroup patterns from this rural African setting temporally map with global strain distribution, suggesting a well-mixed global virus transmission pool of HMPV. Persistence in the local community is characterized by repeated introductions of HMPV variants from the global pool. The factors underlying the declining prevalence of HMPV in this population should be investigated.

**Electronic supplementary material:**

The online version of this article (10.1186/s12879-019-4381-9) contains supplementary material, which is available to authorized users.

## Background

Human metapneumovirus (HMPV) is single-stranded, negative-sense RNA virus with a genome of about 13 kb [1, 2]. The virus belongs to the *Pneumoviridae* family and genus *Metapneumovirus*. HMPV infections occur across all ages with severe disease predominantly occurring in children below 2 years of age and the elderly [3–5]. Since first description in 2001 [5], HMPV has been detected in all continents and its disease prevalence varies widely [6]. Nearly every child by the age of 5 years has been infected by HMPV [5, 7]. Clinical presentation of HMPV infection ranges from mild upper respiratory tract illness to severe lower respiratory tract disease [8] and overlaps with that of other common respiratory viruses especially respiratory syncytial virus (RSV) [9, 10].

HMPV is classified into two major groups, A and B, based on antigenic variation and nucleotide differences in the fusion (F), nucleoprotein (N) and attachment (G) glycoprotein genes [2, 11, 12]. Phylogenetic analyses of F (open reading frame, i.e. ORF) and G (ORF) sequences further divides the two groups into subgroups i.e. A1 and A2 (group A), and B1 and B2 (group B) subgroups [11, 13]. In addition, there has been report of within subgroup A2 division, i.e. presence of two phylogenetically distinct clades A2a and A2b [14]. A few studies have revealed increased heterogeneity of A2, including identification of another distinct clade, provisionally assigned as A2c [15, 16]. Furthermore, two novel clades within subgroup A2 with 180 or 111 nucleotide duplications in the G gene have been detected [17–20]. Geographically, HMPV subgroups have been reported to circulate widely and cluster temporally [21], with the exception of subgroup A1 that has been identified in a few countries [21–23]. There is frequent co-circulation of subgroups with replacement of the predominant subgroup after a period of one or two seasons, although the drivers of this phenomena are unclear [21]. Virus prevalence also varies from year-to-year within the same location [24, 25].

A few studies have reported long-term subgroup circulation patterns of HMPV, necessary for improved epidemiological understanding and, in due course, of potential value to the design and implementation of control measures. In addition, there are a few studies on HMPV in Africa, which bears a high burden of pneumonia morbidity and mortality [26, 27]. In this study, we analysed the surface F and G gene nucleotide sequences collected through paediatric pneumonia surveillance at the Kilifi County Hospital in rural coastal Kenya, from 2007 to 2016, to describe the molecular epidemiology and gain insights into the spread and the evolutionary dynamics of HMPV. The two genes (F and G) code for immunogenic surface proteins that are the targets for vaccine development. G gene is highly variable and is used to assess the diversity of HMPV. Similarly, F gene which is quite conserved, has been used to characterise HMPV and is the gene of target in many molecular diagnostic assays. This report extends our previously published work from the same location for the period 2007–11 [28] and the analysis includes sequences from this previous work.

## Methods

### Study population

The study was conducted at the Kilifi County Hospital (KCH) as part of long-term surveillance aimed at understanding the epidemiology and disease burden of RSV-associated pneumonia cases [29]. KCH, located in coastal Kenya, is a referral hospital serving the population of around 260,000 (circa 2012) within the Kilifi Health and Demographic Surveillance System [30], and beyond in the wider Kilifi County. The population is mainly rural-agrarian. Upon presentation of a child to the paediatric ward at KCH, a detailed medical review is undertaken by the clinician upon which the decision to admit is made. For this study, children (≤ 59 months of age) admitted to the paediatric ward between January 1st 2007 and December 31st 2016 were eligible if they presented with modified WHO defined syndromic severe or very severe pneumonia as previously described [29]. During the period between 2007 and 2009, only admissions arising from residents of Kilifi Health and Demographic Surveillance System (KHDSS) were eligible, whereas in later years, non-KHDSS residents were included. Following written informed consent from the parent or guardian, a nasopharyngeal flocked swab, nasal wash or combination of nasopharyngeal swab and oropharyngeal swab was collected from each child and transferred into viral transport medium for laboratory screening. Ethical approval for the study was obtained from the Kenya Medical Research Institute Scientific and Ethics Review Unit.

### RNA extraction and rRT-PCR

For samples collected from 2007 to 2011 the methods for extraction and detection were as previously described [31, 32]. In brief, for samples collected in 2007 RNA was extracted using Magnapure LC32 total nucleic acid extractor (Roche, Manheim, Germany) and virus screening conducted using the LightCycler Fast Start DNA MasterPLUS Hyb-Probe kit (Roche, Mannheim, Germany) following manufacturer’s instructions. From 2008 to 2011, sample extraction was by MagNaPure LC32 Kit (Roche, Manheim, Germany) or QIAmp Viral RNA Minikit (Qiagen, Germany) and virus detection carried out using a one-step in-house multiplex real time reverse transcription-PCR (rRT-PCR) using a TaqMan probe-based system targeting the fusion (F) gene [28, 32]. Samples with a rRT-PCR cycle threshold (Ct) value < 35 were considered HMPV positive. From 2012 to 2016 total RNA extraction was performed using Qiacube HT automated total nucleic acid extractor (Qiagen, Germany) and virus detection carried out using the same one-step in-house multiplex real time reverse transcription-PCR (rRT-PCR) used for samples from 2008 to 11 [28].

### F and G gene sequencing

HMPV positive samples identified between 2007 and 2011 (*n* = 160) were partially sequenced in the F and G genes, 345 and 606 bp, respectively [28]. For HMPV positive samples identified between 2012 and 2016 (*n* = 114), we amplified full F and G encoding genes, approximately 1620 bp and 700 bp, respectively, using one-step RT-PCR kit (Qiagen, Germany) according to manufacturer’s instructions. RT-PCR and sequencing primers are shown in Additional file 1. Thermocycling conditions for both genes were set at: 50 °C for 30 min, 95 °C for 15 min, 38 cycles of 94 °C for 1 min, 53 °C for 1 min, 72 °C for 1 min, and a final extension of 10 min at 72 °C. HMPV A and B subtype specific primers were used to increase the sensitivity and the performance of G gene amplification. HMPV positive samples were sequenced in both forward and reverse directions using Big dye terminator v1.3 chemistry run on ABI 3130xl instrument [33].

### Sequence analysis and diversity

Sequence reads for the successfully amplified HMPV positives identified between 2012 to 2016 were edited and assembled using Sequencher v5.0.1 (Gene Codes Corporation). The newly assembled F and G genes sequences were deposited in GenBank under accession numbers MH482553 to MH482743. The accession numbers (MH482553 to MH482743) include additional 31 sequences that were detected by different rRT-PCR assay and therefore they were excluded in this analysis. The accession numbers for the excluded sequences (15 for F gene and 16 for G gene) are given in Additional file 2. For subsequent analyses, previously sequenced data for HMPV positives identified between 2007 and 2011 i.e. F (*n* = 123) and G (*n* = 56) genes [28] (accession numbers KT191299 to KT191484) was combined with the newly sequenced data, making a total of 210 sequences for F gene and 118 sequences for G gene used in subsequent analyses. Sequences were aligned with MAFFT v7.220 [34]. Nucleotide and amino acid mean genetic distances within and between subgroups were determined using Kimura-2 parameter model in MEGA v7. 0.2.6 [35]. Further, to assess sequence diversity within epidemics, collated G gene sequences were aligned by epidemic sampling to show changes in the G gene within epidemics.

### Phylogenetic analysis

Contemporaneous sequences (2007 to 2016) were obtained from GenBank for both F and G genes. Sequences had to have a complete overlap in their sequenced portion with the Kilifi sequence data. Duplicate sequences were dropped. A total of 714 F gene sequences and 500 G sequences were retrieved. For phylogenetic construction, sequences were filtered per country using an inhouse ruby script to obtain only unique sequences. Unique sequences were identified as sequences that differ by at least one nucleotide from any other sequence over the sequenced region. Combined global and Kilifi sequences were aligned using MAFFT v7.220. Best fitting nucleotide substitution and site heterogeneity models were estimated in MEGA v7. 0.2.6 and phylogenetic trees inferred using Maximum Likelihood (ML) with 1000 bootstrap replicates. Reference sequences from GenBank (FJ168778 NL/94/01/B2, AY525843 HMPV NL/1/99/B1, AF371337 HMPV NL/1/00/A1, AB503857, AY530095 HMPV JP/240/03/A2b and GQ153651 HMPV CN/gz01/08/A2) were included [36]. A fragment length of 345 bp was analysed for F gene and at least 640 bp for G gene. Subgroups were confirmed if sequences clustered with the reference sequences within a major branch with > 70% bootstrap support on the ML tree. Mean nucleotide genetic distances were also determined to assess sequence similarity between Kilifi and the global data set. To further assess the clustering of HMPV subgroups, ML tree was reconstructed using only full F (1593 bp) gene sequences.

### Evolutionary and selection pressure analysis of Kilfi HMPV viruses

Substitution rates and time to the most recent common ancestor (tMRCA) were determined under uncorrelated lognormal relaxed molecular clock using Bayesian Markov Chain Monte Carlo-based approach implemented in BEAST v1.8.4. Demographic histories of HMPV subgroups were inferred using GMRF Bayesian Skyride model. BEAST analysis was set at 50 million states with sampling every 2500 steps. Selection pressure within F and G genes was analysed by estimating the ratios of non-synonymous to synonymous substitutions (dN/dS = ω), using the codon-based phylogenetic method (CODEML) in PAML v 4.8a. Different site models were employed i.e. M0 (one ratio), M1a (Nearly Neutral), M2a (Positive Selection) and M3 (discrete) model. M0 model calculates a rough average value (ω) distributed across all sites (homogeneous ratios). M1a assumes two categories of sites: conserved sites with ω = 0 and neutral sites with ω = 1. M2a allows for three classes i.e. neutral ω = 1, purifying (0 ≤, ω < 1), and positive selection ω > 1. M3 estimates ω ratios as M2a but using an unconstrained discrete distribution [37, 38]. The models M0 and M1a were used to test for selection over the entire sequenced regions while M2a and M3 tested for positively selected sites. Only sites with posterior probabilities > 95% were considered as positively selected based on Bayes Empirical Bayes method.

### HMPV subgroup and disease severity

Different Kilifi HMPV subgroups identified between 2007 and 2016 were determined. To assess the association between HMPV subgroup and disease severity, we performed Fisher’s exact test. Individuals were defined syndromically as either severe or very severe based on WHO definitions [39]. The clinical definitions have been described previously [28, 29, 31]. A *p*-value of ***≤*** 0.05 was considered to be significant. Statistical analysis was conducted using STATA v13.1 (College Station Texas, USA).

## Results

### HMPV prevalence in paediatric hospital admissions

Between January 2007 and December 2016, 9079 individuals below 60 months of age were admitted to the paediatric wards at KCH with severe or very severe pneumonia. Samples were collected from 6756 (74%) individuals and 274 (4.1%) were determined HMPV positive (Table 1). Decreased HMPV prevalence was recorded in more recent years i.e. from 2014 to 2016 (Table 1). Among the 274 HMPV positive cases, 43.1% (*n* = 118) were in children < 6 months of age and 71.5% (*n* = 196) in children < 2 year of age. Children ≥3 years accounted for only 3.6% of the cases (n = 11) (Table 2).

**Table 1:** Number of samples tested for HMPV and cases positives per year from 2007 to 16 amongst pneumonia paediatric admissions to Kilifi County Hospital, Kenya

Year	Admissions^a^	Eligible^b^	Tested^c^	HMPV Positive	HMPV Prevalence (95% CI)
2007	3506	1174	726	21	2.90 (1.80–4.39)
2008	3204	1009	435	34	7.80 (5.47–10.75)
2009	3633	1105	526	46	8.70 (6.47–11.49)
2010	3091	1090	903	27	3.00 (1.98–4.32)
2011	3005	906	730	32	4.40 (3.02–6.13)
2012	2715	863	943	50	5.30 (3.96–6.93)
2013	2074	566	540	28	5.20 (3.47–7.41)
2014	2947	797	695	18	2.60 (1.54–4.06)
2015	3029	793	658	8	1.20 (0.53–2.38)
2016	2803	776	600	10	1.70 (0.80–3.04)
Overall	30,007	9079	6756	274	4.10

Abbreviations: *CI* Confidence intervals, *HMPV* Human metapneumovirus

a. Admissions under 5 years of age; b. Cases of severe or very severe pneumonia amongst the admissions [29]; c. Samples eligible for testing from the pneumonia admissions included only residents from within the Kilifi Health and Demographic Surveillance System (KHDSS) for years 2007–9, and residents or non-residents for other years

**Table 2:** HMPV positives stratified by age of patients admitted to Kilifi County Hospital, Kenya 2007–2016

Age Categories	Frequency	Percent
0-2 M	51	18.61
3-5 M	67	24.45
6-8 M	43	15.69
9-11 M	35	12.77
12-17 M	20	7.30
18–23	20	7.30
25-35 M	27	9.85
36 + M	11	4.01
Total	274	100

### HMPV subgroup assignment

**Fig. 1:**
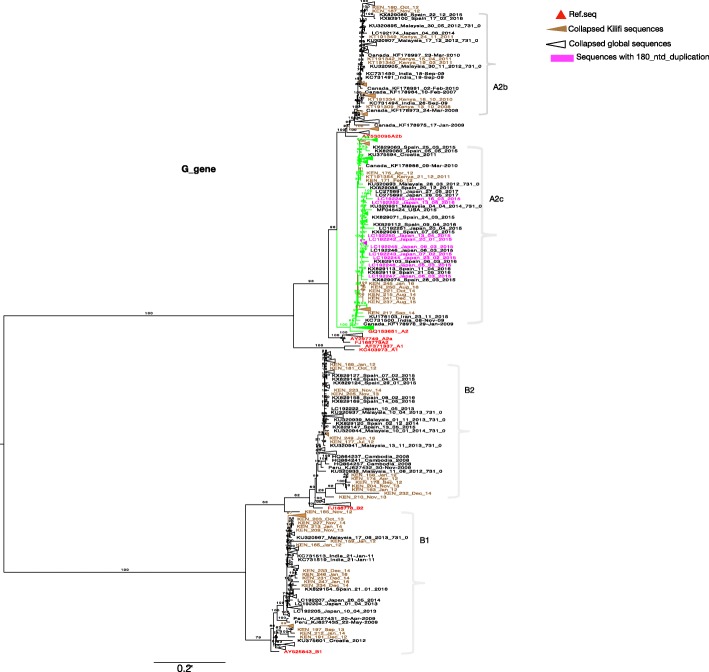
A ML phylogenetic tree of 600 Gene sequences constructed using Kilifi sequences and sequences obtained from GenBank, collected from 2007 to 2016. Sequences were subtyped using references sequences retrieved from GenBank. Reference sequences colored in red. The numbers next to branches indicate the bootstrap values. Subgroups were confirmed if sequences clustered with the reference sequences within a major branch with > 70% bootstrap support. Taxa colored in magenta represent strains that have 180 nucleotide duplications retrieved from GenBank. Kilifi sequences are colored in brown

**Fig. 2:**
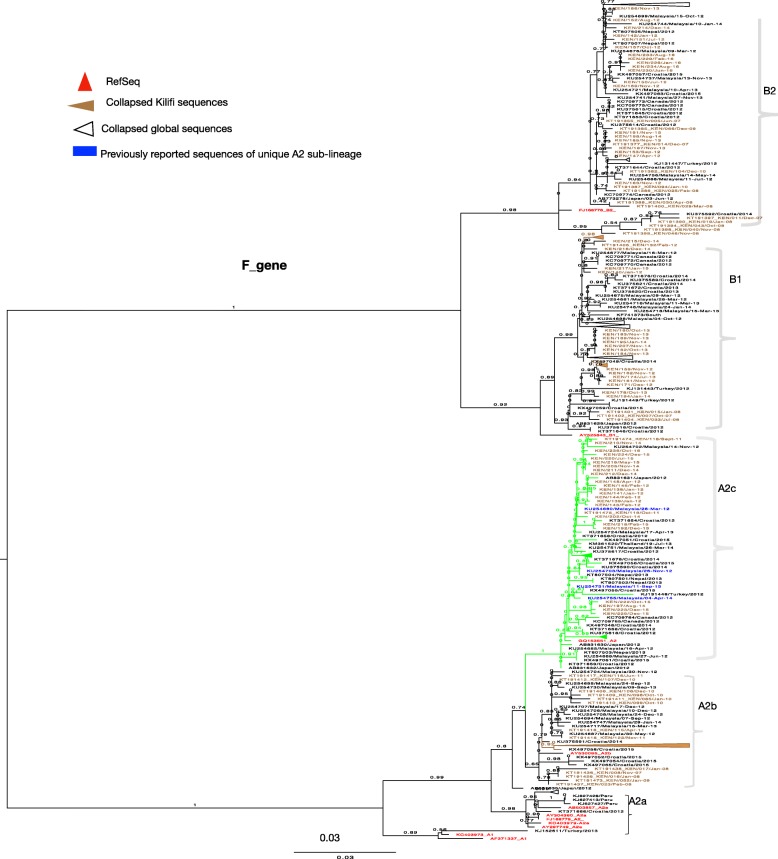
A ML phylogenetic tree of 563 unique F Gene sequences constructed using Kilifi sequences and sequences obtained from GenBank, collected from 2007 to 2016. Sequences were subtyped using references sequences retrieved from GenBank. Reference sequences colored in red. The numbers next to branches indicate the bootstrap values. Subgroups were confirmed if sequences clustered with the reference sequences within a major branch with > 70% bootstrap support. Taxa colored in blue represent sequences of previously reported unique sub-lineage of A2, retrieved from GenBank. Kilifi sequences are colored in brown

Phylogenetic analysis (F and G gene) of Kilifi, global and the reference sequences showed clustering of HMPV viruses into subgroups A2, B1 and B2. Subgroup A1 was not observed. Subgroup A2 sequences further clustered into 3 clades, the two known clades A2a and A2b, and into the unique clade A2c supported by strong bootstrap values (Figs. 1 and 2). The recently identified unique HMPV A2 strain, which possess 180 duplication in G gene clustered within A2c clade (Fig. 1) and was not observed in the Kilifi sequences. Based on F gene phylogeny constructed using full length coding regions, similar clades and sub-groups were also observed i.e. A2a, A2b, A2c, B1 and B2 (Additional file 3).

The prevalence of group A was 51.7% (106/205) and group B 48.3% (99/205)**.** All the group A Kilifi sequences clustered within clade A2b (84/106, 79.2%) and within the unique clade A2c (22/106, 20.8%). No A1 or A2a sequences were observed. Within group B, 48.5% (48/99) of the sequences clustered within subgroup B1 and 51.5% (51/99) of the sequences clustered within subgroup B2.

### Temporal occurrence of HMPV in Kilifi

**Fig. 3:**
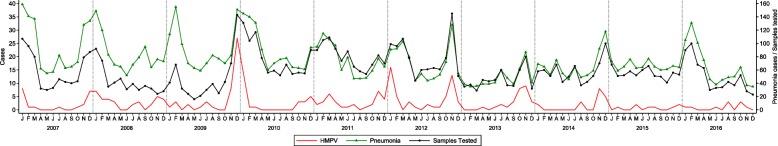
Temporal distribution of HMPV cases identified monthly from surveillance of severe and very severe pneumonia cases aged under 5 years admitted to Kilifi County Hospital, Kenya 2007–2016. Number pneumonia cases and number tested are shown on the secondary Y axis and HMPV cases detected shown on the primary Y axis. Samples eligible for testing from the pneumonia admissions included only residents from within the Kilifi Health and Demographic Surveillance System (KHDSS) for years 2007–9, and residents or non-residents for other years

During the 10-year period of surveillance described here, HMPV infections occurred in a seasonal pattern, mainly between the month of October of one year and April of the following year, Fig. 3, which was used to define an epidemic year, i.e. starting September of one year through August of the following year. Thus, subsequent analyses were done by epidemic year. Multiple subgroups co-circulated within a single epidemic year from 2007 to 2016, Fig. 4b. There was a shift in dominance from A2b (2007 to 2011) to B1 (2012 to 2014) with subsequent rise of the A2c in recent years. Notably, B2 subgroups remained persistent but non-dominant. A2b viruses circulated only until November 2012.

**Fig. 4:**
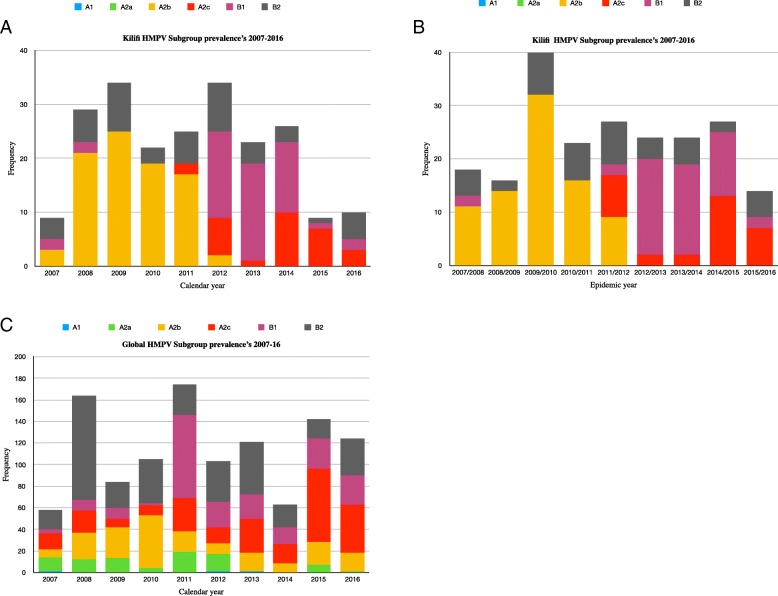
HMPV subgroup prevalence patterns Kilifi versus Global from 2007 to 2016. Panel **a**, Kilifi subgroup temporal patterns derived from Kilifi sequence data determined by calendar year. Panel **b**, Kilifi subgroup temporal patterns determined by epidemic year. Panel **c**, global subgroup temporal patterns determined from combined 714 F sequences and 500 G sequences retrieved from GenBank

HMPV subgroup prevalence and dominance in Kilifi was compared to other countries around the world. This was based on analysis of 714 F gene sequences from 18 different countries and 500 G gene sequences from 15 countries retrieved from GenBank (Additional file 2). General agreement in subgroup prevalence patterns was observed for Kilifi and global data sets, the predominance of A2b and B2 in 2007–2010 and subsequent rise of B1 and A2c in years that followed, Fig. 4. Notably, at finer levels there were differences in subgroup temporal occurrence. Globally, clades A2b, A2c and subgroup B1 persisted over the 9 years. In contrast, in Kilifi only subgroup B2 persisted over the 9 years. The dominance of B1 strains in 2011 was also evident globally. A1 and A2a were the least commonly detected subgroups globally and not observed in Kilifi.

### Phylogenetic analysis of Kilifi HMPV viruses

**Fig. 5:**
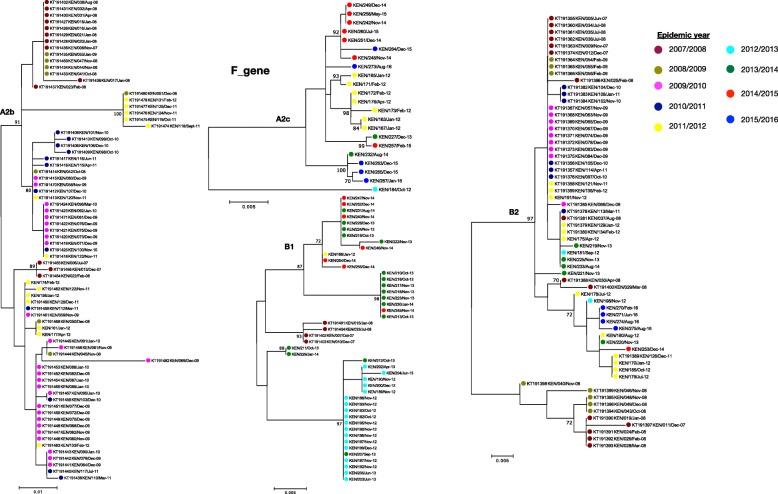
ML phylogenies by subgroup for F gene Kilifi sequences colored by epidemic, from 2007 to 2016. The numbers next to branches indicate the bootstrap values, a branch with > 70% bootstrap value was considered as a major branch

**Fig. 6:**
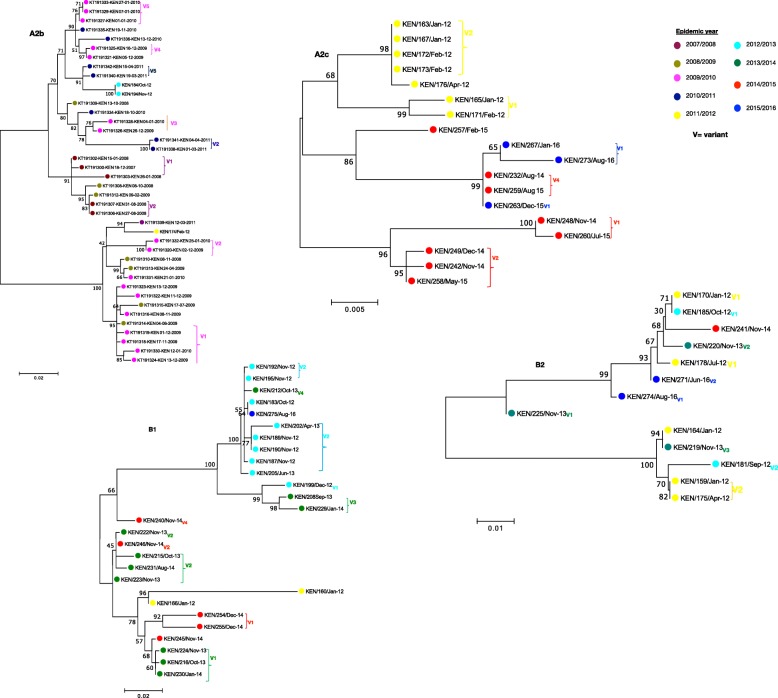
ML phylogenies by subgroup for G gene Kilifi sequences colored by epidemic, from 2007 to 2016. The numbers next to branches indicate the bootstrap values, a branch with > 70% bootstrap value was considered as a major branch. Different variants observed within an epidemic are indicated next to branches colored by epidemic year. Cut off > = 70% bootstrap support was considered significant for variant assignment. The nucleotide differences between the variants are shown in Additional files 4 and 5

Based on maximum likelihood phylogenies of F and G genes, sequences from consecutive epidemics largely clustered together. However, across multiple epidemics sequences from the earlier years clustered together away from those in recent years, Figs. 5 and 6**.** Further analysis of G gene sequences aligned temporally, revealed circulation of multiple variants of a single subgroup in a single epidemic year (see Additional files 4 and 5)**.** Each variant differed by at least five nucleotides (Additional files 4 and 5) and clustered into separate clades, Fig. 6**.** In comparison to contemporaneous viruses (714 F gene sequences and 500 G gene sequences) (Additional file 2), viruses from Kilifi and those from other locations were highly similar, with an average nucleotide identity of 97.1 and 95.6% (in F gene), and 90.4 and 85.9% (in G gene) for groups A and B, respectively. In phylogenies of F and G, combining contemporaneous and Kilifi sequence data, the Kilifi sequences did not cluster separately from others but interspersed with sequences from Canada, Spain, Malaysia, Japan, USA, India and Croatia **(**Figs. 1 and 2), and similarly, those viruses collected from earlier years in Kilifi clustered away from those in recent years.

### F gene sequence analysis

Due to partial gene sequencing (345 bp) of the previously reported F gene Kilifi sequences retrieved from GenBank [28], combined Kilifi sequences were trimmed to 345 bp. The F gene sequences were highly conserved both at the nucleotide (nt) and amino acid (aa) level with overall mean sequence identity of 89% (nt) and 97% (aa). Within the individual subgroups, the nucleotide and amino acid sequences were more conserved: there was 100% aa sequence similarity within clades A2b, A2c and subgroup B1, and 99.9% within B2 subgroup, and the mean nucleotide identities were 98.2% within B1, 99.2% within A2c, 98.3% within both A2b and B2. The evolutionary rates for F gene were estimated at **1.220× 10**^**− 3**^ (95% highest posterior density (HPD) = 1.317× 10^− 3^ to 5.518× 10^− 3^), **1.857× 10**^**− 3**^ (95% HPD = 1.111× 10–3 to 2.289× 10^− 3^), **1.242× 10**^**− 3**^ (95%HPD = 8.303× 10^− 4^ to 1.689× 10^− 3^) and **1.050× 10**^**− 3**^ (95%HPD = 5.937× 10^− 4^ to 1.600× 10^− 3^), nucleotide substitutions/site/year for A2c, A2b, B1 and B2, respectively. Selection pressure analysis of the F gene showed overall mean dN/dS ratio of ω = 0.10, ω = 0.15, ω = 0.01 and ω = 0.12 for A2c, A2b, B1 and B2, respectively (see Additional file 6). Using M2a and M3 models, no codon sites were identified as positively selected among the four types (sub-lineage A2b, sub-lineage A2c, B1 and B2 subgroups). (Additional file 6).

### G gene sequence analysis (Kilifi)

G gene sequences were less conserved compared to F gene sequences with overall mean sequence identity of 73% (nt) and 56% (aa). However, within subgroups the sequences were highly conserved: the mean identities were estimated at 97% (nt) and 96% (aa) for clade A2c, 93% (nt) and 89% (aa) for clade A2b, 94% (nt) and 90% (aa) for B1, 92%(nt) and 87% for B2 subgroups. The deduced G gene amino acid sequences differed in lengths among subgroups: clade A2c (220aa), clade A2b (218 and 229aa), B2 (238aa) and B1 (232 and 242aa) long. The varying protein lengths for clade A2b and B1 subgroups were due to changes in stop codon positions. The evolutionary rates for the G gene were estimated at **3.420× 10**^**− 3**^ (95% HPD interval = 1.317× 10^− 3^ to 5.518× 10^− 3^), **8.022× 10**^**− 3**^ (95% HPD = 5.893× 10^− 3^ to 1.060× 10^− 2^) and **9.570× 10**^**− 3**^ (95% HPD = 5.911× 10^− 3^ to 1.33× 10^− 2^) nucleotide substitutions/site/year for A2c, A2b and B1, respectively. Evolutionary rate estimates for subgroup B2 viruses were not determined, effective sample size (ESS) for all parameters failed to reach the cut-off (200) required for confirmation of convergence in BEAST analysis. Among all the subgroups, A2c exhibited significantly lower substitution rates within the G gene (95% HPD = 1.317× 10^− 3^ to 5.518× 10^− 3^). The estimated times of the most common ancestor of the individual subgroups dated to recent origin in both F and G genes, i.e., 2009 (95% HPD = 2005–2011) for clade A2c sequences, 2008 (95% HPD = 2006–2009) for clade A2b, 2005 (95% HPD = 2002–2007) for B1 and 2001(95% HPD = 1997–2005) for B2 sequences. The estimated mean dN/dS ratio (ω) was < 1 for A2b (ω = 0.57), A2c (ω = 0.38) and B1(ω = 0.78). For the subgroup B2, a higher average dN/dS ratio (ω = 1.21) was determined for G gene, indicating a signal of positive selection (Additional file 6). Using M2a and M3 models, 3 positively selected sites were identified within A2c (17, 24, 28, and) and A2b (87, 123 and 135), 1 site (96) in B1 and 2 sites (12,127) within B2 sequences (Additional file 6).

### HMPV subgroup and disease severity

We did not observe a statistically significant association between HMPV subgroup and pneumonia severity ( *p*-value = 0.264) (Table 3).

**Table 3:** Association between different HMPV subgroups and disease severity

Subgroup		WHO Pneumonia Status		Total
		Severe	Very severe	
A2b	n	71	13	84
	%	84.52	15.48	100
A2c	n	20	2	22
	%	90.91	9.09	100
B1	n	35	13	48
	%	72.92	27.08	100
B2	n	41	10	51
	%	80.39	19.61	100
	n			
Total	%	167	38	205
		81.46	18.54	100
				*p*-value = 0.264

## Discussion

There remains limited information on long-term circulation patterns and prevalence of HMPV in Africa. This study reports HMPV subtype incidence and molecular evolution in coastal Kenya over a 10-year period of surveillance (2007 to 2016). HMPV incidence in children under 5 years of age varied annually ranging from 1.2 to 8.7% and 71.5% of HMPV infections were in children aged < 2 years. Wide variation in annual prevalence has been reported elsewhere from long-term studies of acute respiratory infection (ARI) aetiology in children: in Germany, HMPV prevalence over 10 years of surveillance varied between 1.4 and 32.8% [21], and in Italy, the prevalence fluctuated from 37 to 7% and then to 43% over a 3 year period [24]. To date the cause of the varied prevalence and whether there is difference in disease severity among HMPV subgroups remains unclear. Different studies report contrasting results on the association of disease severity among the HMPV subgroups [10, 40]. In this study, no significant association was found between subgroup and disease severity or clinical presentation. Further studies are necessary to evaluate whether changes in subgroup are associated with changes in disease severity and prevalence. Climatic factors such as temperature, rainfall and relative humidity have also been shown to influence HMPV activity in the tropics [23]. In this study, seasonal increase in cases tended to coincide with low rainfall, lower relative humidity and higher temperatures. Further investigation is required to determine whether changes in such environmental factors influence HMPV activity.

Kilifi HMPV subgroup prevalence patterns partly mirrored global patterns, there was predominance of A2b and B2 in 2007–2010 and subsequent rise of B1 and A2c in years that followed. B2 viruses persisted over the 9 years. A1 and A2a were the least commonly detected subgroups globally and this could probably explain why they were not detected in Kilifi. In contrast to continued circulation of clade A2b [18], in Kilifi, A2b circulated from 2007 to November 2012 and no longer. Occurrence of the clade A2c [41] has increased in Kilifi in recent years, as similarly reported in Bangladesh and Croatia [15, 16], suggestive of global spread of HMPV variants. Based on F and G phylogenies, some of previously reported sequences from Europe, Asia and USA that either had not been sub-grouped or assigned to clade A2b, clustered within A2c, consistent with findings from a previous report [15]. Similarly, previously reported sequences of the unique A2 clade reported in Malaysia [23] also clustered in this clade (A2c). Recently, two novel HMPV A2 strains with 180 or 111 nucleotide duplication in the G gene have also been reported [17, 20]. These strains were not observed in Kilifi. In the phylogenetic trees they clustered within A2c together with other A2c sequences without duplication including Kilifi A2c strains. Our findings reaffirm the circulation of A2c clade and point out the need for proper methods for classification of HMPV lineages. The replacement patterns of HMPV subgroups could be attributed to subgroup-specific herd immunity offering protection against circulating homologous strains but not against heterologous strains. This observation is not unique to Kilifi, as similar patterns were reported from long-term surveillance studies in Germany and France [21, 42]. Consistent with earlier studies, G gene exhibited higher rates of evolution compared to F gene [2]. F gene is more conserved and our findings concur with previous studies on F protein diversity [43]. The rates of G gene evolution were in the range of 3.42× 10^− 3^ to 9.57× 10^− 3^, similar to findings from previous studies [44, 45]. Among the subgroups, the rates of evolution for A2b and B1 were at least 2 fold higher than other subgroups, this concurs with the findings from a similar study [44]. In general, higher polymorphism has been reported in HMPV G gene and further within the subgroups resulting from events such as changes in alternative transcription termination codons and insertions [46]. This could explain the differences in evolution rates. Further investigations are required to give more insights on the differences in evolution rates and its implication. Compared to other *Pneumoviruses*, such as human respiratory syncytial virus (HRSV), HMPV G gene has been shown to have higher rates of evolution [45]. Our estimates show comparable results, the rates for HMPV A2b and B1 were 3 to 4 times higher than those reported for HRSV (3.58× 10^− 3^, 95% HPD = 3.04× 10^− 3^ to 4.16× 10^− 3^) [47]. The difference in the rates of evolution can be linked to strong association between neutralizing epitopes and positively selected sites reported for RSV G gene [48]. In contrast to the case for other paramyxoviruses, such as RSV, the HMPV G protein is not a major neutralizing or protective antigen [49].

Temporal analysis revealed circulation of multiple subgroups within a single epidemic. Further analysis of the G glycoprotein encoding gene revealed circulation of multiple variants for a single subgroup within an epidemic. Multiple variants observed might be attributed to high rate of G gene evolution (substitutions/site/year) resulting in diversification in situ during a season. However, the level of variation observed within a subgroup within epidemics was greater that would be expected from in situ diversification, implying multiple variant introductions of a subgroup during single epidemic years.

As is the norm for HMPV detection in respiratory samples, we used molecular PCR-based diagnostics [6]. Recent studies have shown that mutations at primer and probe binding sites can lead to false negative diagnostic results and hence underestimation of disease burden [50, 51]. Evaluation of the in-house diagnostic rRT-PCR assay would be important to determine whether there are any missed variants/subgroups and whether this can be associated with the apparent gradual decline in HMPV prevalence observed in the current dataset. There is also some evidence that reduction in bacterial pneumonia (e.g. *S. pneumoniae*), as has been seen in this coastal location over this study period [52], results in a reduction in viral pneumonia [53, 54]. Future investigations will be necessary to give more insight.

The analysed Kilifi F and G protein encoding genes were generally determined to be under purifying selection pressure, which drives RNA virus evolution by purifying the deleterious mutations due to RNA replication errors [55]. However, for the B2 subgroup, a higher dN/dS ratio was observed in G gene sequences suggestive of diversifying selection within B2 viruses. The distinct diversifying selection and persistence of the B2 viruses observed requires further investigation. In this study, our F and G genes sequence analyses were based on partial gene lengths, 345 bp and 640 bp, respectively. Therefore, our results on the genetic distance estimates, evolutionary and selection pressure analysis should be interpreted with caution. The partial lengths may have reduced our potential to discriminate genetic clusters, a possible explanation for higher sequence similarity observed between and within the subgroups for F gene. Overall, although our sequence analyses were limited to partial lengths, the newly designed F and G genes primers allowed full length sequencing of the two genes for newly generated data. In addition, the newly designed G gene subtype specific primers allowed sequencing of all HMPV subtypes and significantly improved G PCR recovery by two-fold compared to previously reported assay. This improved the study power to characterise the different circulating HMPV variants. Overall, we successfully collected clinical samples from 74% (6756/9076) of the study enrolled participants and characterised 75% (205/274) of the HMPV viruses identified. In this study we failed to collect samples from 24% of the eligibles. We have previously reported similar results, which results from refusals and difficulty in collection of nasal specimens from children with very severe disease [29]. Hence, if some HMPV variants are associated with disease severity there may be bias in the prevalence and variant composition estimates. However, the proportion not collected shows no systematic change over time. In addition, the proportion of very severe pneumonia cases was high among sampled eligibles compared to unsampled eligibles. Therefore, it’s unlikely there was any bias in the prevalence estimates of HMPV groups or subgroups.

Our analyses on HMPV epidemiology in Kilifi were limited to two viral genes only. Whole genome sequencing might give more insights into transmission, HMPV subgroup characterisation and molecular evolution. Our estimation and inference on the association between HMPV subgroup and disease severity was biased to in-patient surveillance data, and therefore future studies should include outpatient surveillance data. In addition, inpatient surveillance and sampling of HMPV infections might not be representative of the full variant population circulating in this community and not correctly reflect incidence and prevalence, as HMPV infections have also been reported in outpatient settings [56]. Future studies across different locations in Kenya and in Africa will be important for tracing the introduction and transmission patterns of the virus.

## Conclusions

In conclusion, this report shows HMPV activity characterised by marked annual variation in occurrence, and in subgroup prevalence patterns (reflecting those in other locations globally,) gradual replacement of subgroups over time, and multiple circulating variants each epidemic varying year to year. This suggests, widescale and rapid circulation of HMPV subgroups, seasonal epidemics resulting from multiple introductions rather than single source, and possible evidence of herd immunity replacement at the subgroup and within-subgroup cluster level.

## Additional files

Additional file 1: Newly designed F and G gene PCR and sequencing primers. (DOCX 15 kb)

Additional file 2: Panel A and B, GenBank accession numbers for global contemporaneous F (714) and G (500) gene sequences retrieved from GenBank**.** Panel C, GenBank accession numbers for Kilifi sequences detected by alternative rt-PCR assay. (CSV 11 kb)

Additional file 3: Phylogenetic analysis of Kilifi sequences and sequences retrieved from GenBank constructed using only full F gene sequences (1593 bp) to further asses the clustering of HMPV subgroups. A total 185 full F sequences were used. Sequences were subtyped using references sequences retrieved from GenBank. Reference sequences colored in red. The numbers next to branches indicate the bootstrap values, a branch with > 70% bootstrap value was considered as a major branch. (PDF 44 kb)

Additional file 4: Changes in G gene nucleotide sequences for sequences collected from Kilifi. Sequences were aligned by subgroup and by epidemic year for Clade A2b and A2c. Nucleotide differences were determined and indicated by vertical colored bars. Orange red represent Adenine (A), Crimson represent Thymine (T), Indigo represents Guanine (G) and slate blue represent Cytosine (C) nucleotide. (PDF 261 kb)

Additional file 5: Changes in G gene nucleotide sequences for sequences collected from Kilifi. Sequences were aligned by subgroup and by epidemic year for subgroups B1 and B2. Nucleotide differences were determined and indicated by vertical colored bars. Orange red represent Adenine (A), Crimson represent Thymine (T), Indigo represents Guanine (G) and slate blue represent Cytosine (C) nucleotide. (PDF 167 kb)

Additional file 6: Parameter estimates, dN/dS ratios, positively selected sites and log likelihood scores for F and G gene nucleotide sequences for the HMPV subgroups identified between 2007 and 2016. (PDF 48 kb)

## Data Availability

All data generated or analysed during this study has been deposited to the Virus Epidemiology and Control (VEC), KEMRI-Wellcome Trust Research Programme, data server under the DOI: https://dataverse.harvard.edu/dataset.xhtml?persistentId=doi:10.7910/DVN/MJPRLV.

## Abbreviations

ARI: Acute respiratory infections
HMPV: Human metapneumovirus
HPD: Highest posterior density
KCH: Kilifi County Hospital
KHDSS: Kilifi Health and Demographic Surveillance System
RNA: Ribonucleic acid
rRT-PCR: Real time Reverse Transcription Polymerase Chain Reaction
tMRCA: Time to the most recent common ancestor
