# Supplementary material for: Human metapneumovirus prevalence and patterns of subgroup persistence identified through surveillance of pediatric pneumonia hospital admissions in coastal Kenya, 2007–2016
Source: BMC Infect Dis. 2019 Aug 30;19:757. doi: 10.1186/s12879-019-4381-9 (PMC6716807; doi:10.1186/s12879-019-4381-9)
Supplement: Supplementary file 1 — Newly designed F and G gene PCR and sequencing primers. (DOCX 15 kb) [file 12879_2019_4381_MOESM1_ESM.docx]

**Table S1: Newly designed F and G gene PCR and sequencing primers**

|  | **Primer name** |  | **Sequence** | **Gene** | **Position** | **Polarity** | **Subgroup** |
| --- | --- | --- | --- | --- | --- | --- | --- |
| **1** | 11F | PCR + sequencing | AAAATGTCTTGGAAAGTGRTGATY | F | (3,064 -> 3,087) | + | AB |
| **2** | 293F | Sequencing | CAAATTGARAATCCCAGACAATCWAG | F | (3,345 -> 3,370) | + | AB |
| **3** | 587F | Sequencing | AGCTTCAGTCARTTCAACAGAAGR | F | (3,639 -> 3,662) | + | AB |
| **4** | 973F | Sequencing | CCCAAATGARAARGACTGYGAAAC | F | (4,025 -> 4,048) | + | AB |
| **5** | 610R | Sequencing | YCTTCTGTTGAAYTGACTGAAGCT | F | (3,662 -> 3,639) | - | AB |
| **6** | 996R | Sequencing | GTTTCRCAGTCYTTYTCATTTGGG | F | (4,048  -> 4,025) | - | AB |
| **7** | M30R | PCR + sequencing | GGAGCYTTRCGAGACATKATGATT | F | (4,549  -> 4,572) | - | AB |
| **8** | 13F | PCR + sequencing | GTRGAGAACATTCGAGCAATAGACA | G | (6,244  -> 6,268) | + | A |
| **9** | 264F | Sequencing | TCCAAACTCACAGCATCCAAC | G | (6,495  -> 6,515) | + | A |
| **10** | 1163R | PCR + sequencing | AGGGAGATAGACATTAACAGTGGA | G | (7,151  -> 7,174) | - | A |
| **11** | 2F | PCR + sequencing | TGGAAGTAAGAGTGGAGAACATTC | G | (6,213  -> 6,236) | + | B |
| **12** | 222F | Sequencing | YAARAAGACCCCAATGACCTC | G | (6,433  -> 6,453) | + | B |
| **13** | 718R | Sequencing | ACTACTTGGATGAGATACCTGTGT | G | (6,897 -> 6,874) | - | B |
| **14** | 1098R | PCR + sequencing | TGACTGCATTTCTAAGCCTTACAT | G | (7,277  -> 7,254) | - | B |
