# Supplementary material for: Human metapneumovirus prevalence and patterns of subgroup persistence identified through surveillance of pediatric pneumonia hospital admissions in coastal Kenya, 2007–2016
Source: BMC Infect Dis. 2019 Aug 30;19:757. doi: 10.1186/s12879-019-4381-9 (PMC6716807; doi:10.1186/s12879-019-4381-9)
Supplement: Supplementary file 3 — Phylogenetic analysis of Kilifi sequences and sequences retrieved from GenBank constructed using only full F gene sequences (1593 bp) to further asses the clustering of HMPV subgroups. A total 185 full F sequences were used. Sequences were subtyped using references sequences retrieved from GenBank. Reference sequences colored in red. The numbers next to branches indicate the bootstrap values, a branch with > 70% bootstrap value was considered as a major branch. (PDF 44 kb) [file 12879_2019_4381_MOESM3_ESM.pdf]

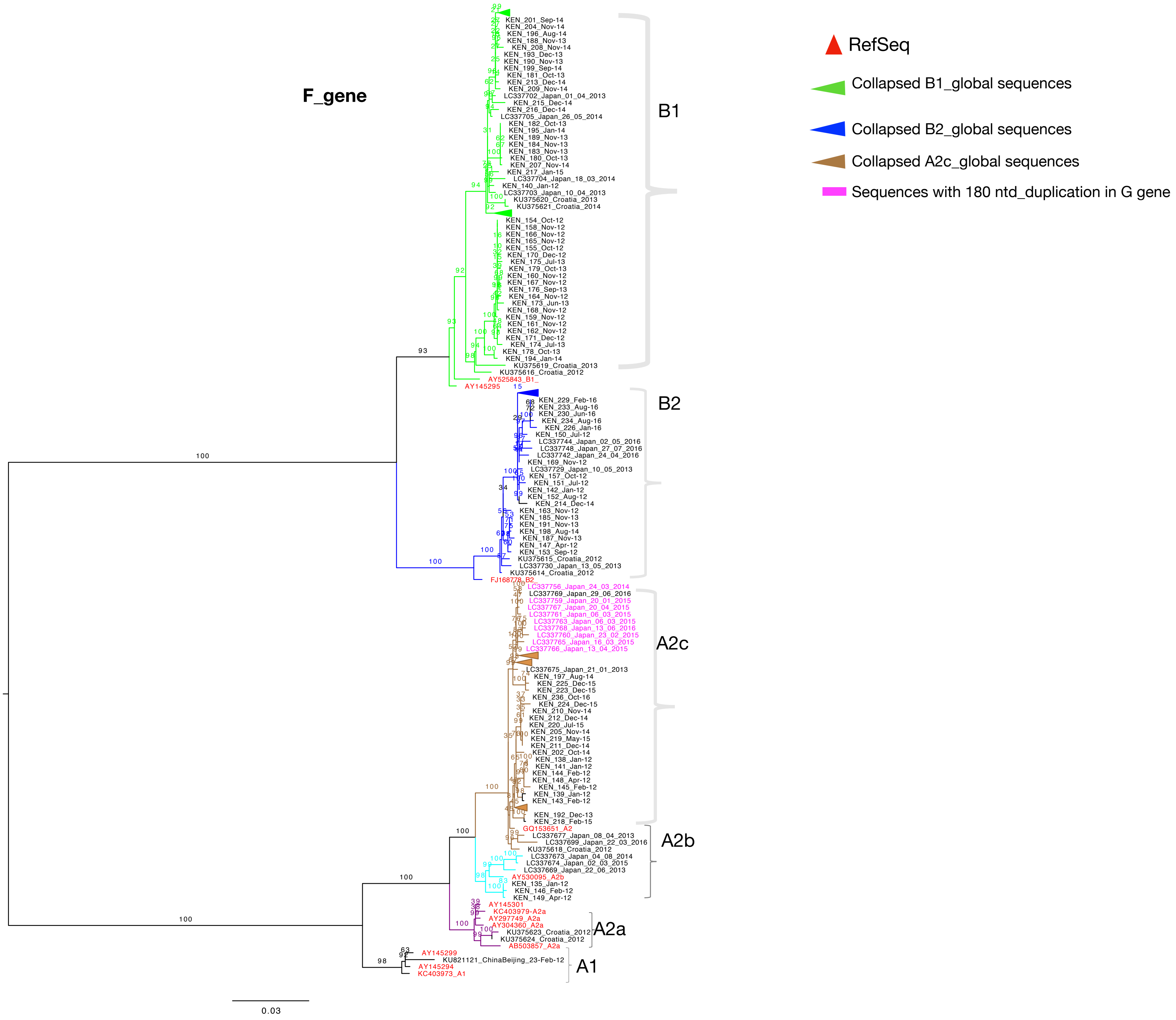
F_gene
RefSeq
Collapsed B1_global sequences
Collapsed B2_global sequences
Collapsed A2c_global sequences
Sequences with 180 ntd_duplication in G gene
B1
B2
A2c
A2b
A2a
A1
KEN_201_Sep-14
KEN_204_Nov-14
KEN_196_Aug-14
KEN_188_Nov-13
KEN_208_Nov-14
KEN_193_Dec-13
KEN_190_Nov-13
KEN_199_Sep-14
KEN_181_Oct-13
KEN_213_Dec-14
KEN_209_Nov-14
LC337702_Japan_01_04_2013
KEN_215_Dec-14
KEN_216_Dec-14
LC337705_Japan_26_05_2014
KEN_182_Oct-13
KEN_195_Jan-14
KEN_189_Nov-13
KEN_184_Nov-13
KEN_183_Nov-13
KEN_180_Oct-13
KEN_207_Nov-14
KEN_217_Jan-15
LC337704_Japan_18_03_2014
KEN_140_Jan-12
LC337703_Japan_10_04_2013
KU375620_Croatia_2013
KU375621_Croatia_2014
KEN_154_Oct-12
KEN_158_Nov-12
KEN_166_Nov-12
KEN_165_Nov-12
KEN_155_Oct-12
KEN_170_Dec-12
KEN_175_Jul-13
KEN_179_Oct-13
KEN_160_Nov-12
KEN_167_Nov-12
KEN_176_Sep-13
KEN_164_Nov-12
KEN_173_Jun-13
KEN_168_Nov-12
KEN_159_Nov-12
KEN_161_Nov-12
KEN_162_Nov-12
KEN_171_Dec-12
KEN_174_Jul-13
KEN_178_Oct-13
KEN_194_Jan-14
KU375619_Croatia_2013
KU375616_Croatia_2012
AY525843_B1_
AY145295
KEN_229_Feb-16
KEN_233_Aug-16
KEN_230_Jun-16
KEN_234_Aug-16
KEN_226_Jan-16
KEN_150_Jul-12
LC337744_Japan_02_05_2016
LC337748_Japan_27_07_2016
LC337742_Japan_24_04_2016
KEN_169_Nov-12
LC337729_Japan_10_05_2013
KEN_157_Oct-12
KEN_151_Jul-12
KEN_142_Jan-12
KEN_152_Aug-12
KEN_214_Dec-14
KEN_163_Nov-12
KEN_185_Nov-13
KEN_191_Nov-13
KEN_198_Aug-14
KEN_187_Nov-13
KEN_147_Apr-12
KEN_153_Sep-12
KU375615_Croatia_2012
LC337730_Japan_13_05_2013
KU375614_Croatia_2012
FJ168778_B2
LC337756_Japan_24_03_2014
LC337769_Japan_29_06_2016
LC337759_Japan_20_01_2015
LC337767_Japan_20_04_2015
LC337761_Japan_06_03_2015
LC337763_Japan_06_03_2015
LC337768_Japan_13_06_2016
LC337760_Japan_23_02_2015
LC337765_Japan_16_03_2015
LC337766_Japan_13_04_2015
LC337675_Japan_21_01_2013
KEN_197_Aug-14
KEN_225_Dec-15
KEN_223_Dec-15
KEN_236_Oct-16
KEN_224_Dec-15
KEN_210_Nov-14
KEN_212_Dec-14
KEN_220_Jul-15
KEN_205_Nov-14
KEN_219_May-15
KEN_211_Dec-14
KEN_202_Oct-14
KEN_138_Jan-12
KEN_141_Jan-12
KEN_144_Feb-12
KEN_148_Apr-12
KEN_145_Feb-12
KEN_139_Jan-12
KEN_143_Feb-12
KEN_192_Dec-13
KEN_218_Feb-15
GQ153651_A2
LC337677_Japan_08_04_2013
LC337699_Japan_22_03_2016
KU375618_Croatia_2012
LC337673_Japan_04_08_2014
LC337674_Japan_02_03_2015
LC337669_Japan_22_06_2013
AY530095_A2b
KEN_135_Jan-12
KEN_146_Feb-12
KEN_149_Apr-12
AY145301
KC403979-A2a
AY297749_A2a
AY304360_A2a
KU375623_Croatia_2012
KU375624_Croatia_2012
AB503857_A2a
AY145299
KU821121_ChinaBeijing_23-Feb-12
AY145294
KC403973_A1
0.03
