# Supplementary figures and images for: Human metapneumovirus prevalence and patterns of subgroup persistence identified through surveillance of pediatric pneumonia hospital admissions in coastal Kenya, 2007–2016
Source: BMC Infect Dis. 2019 Aug 30;19:757. doi: 10.1186/s12879-019-4381-9 (PMC6716807; doi:10.1186/s12879-019-4381-9)

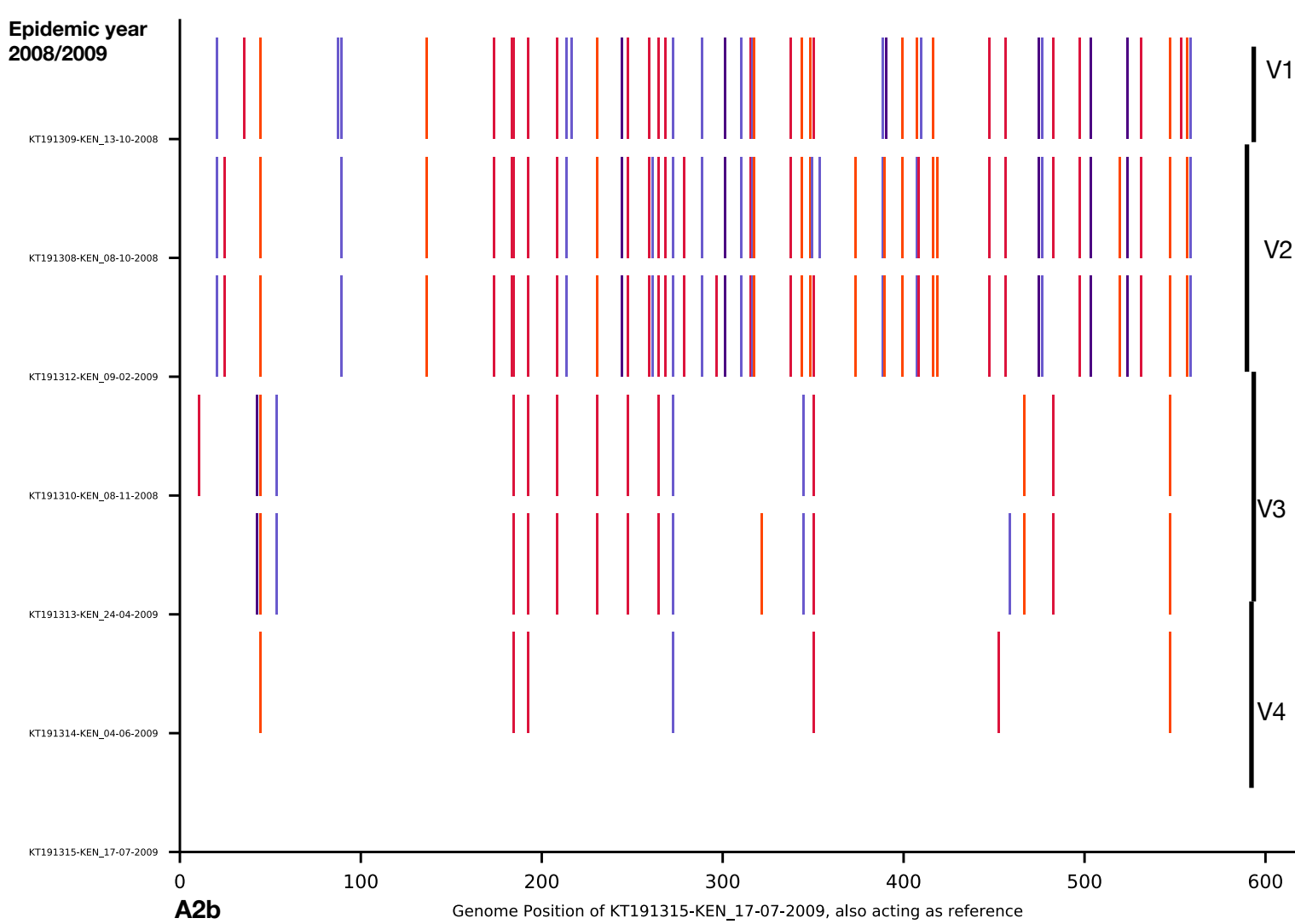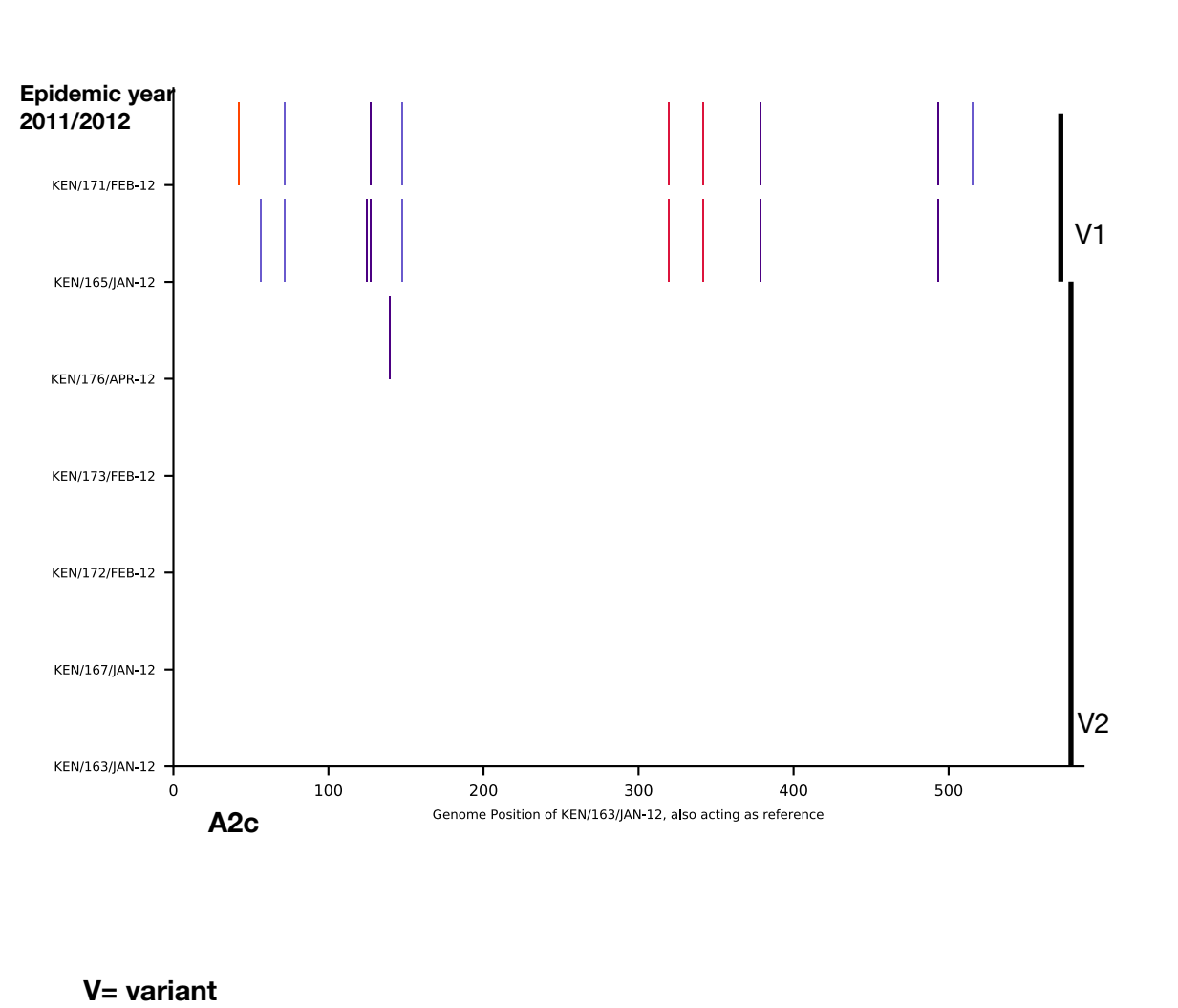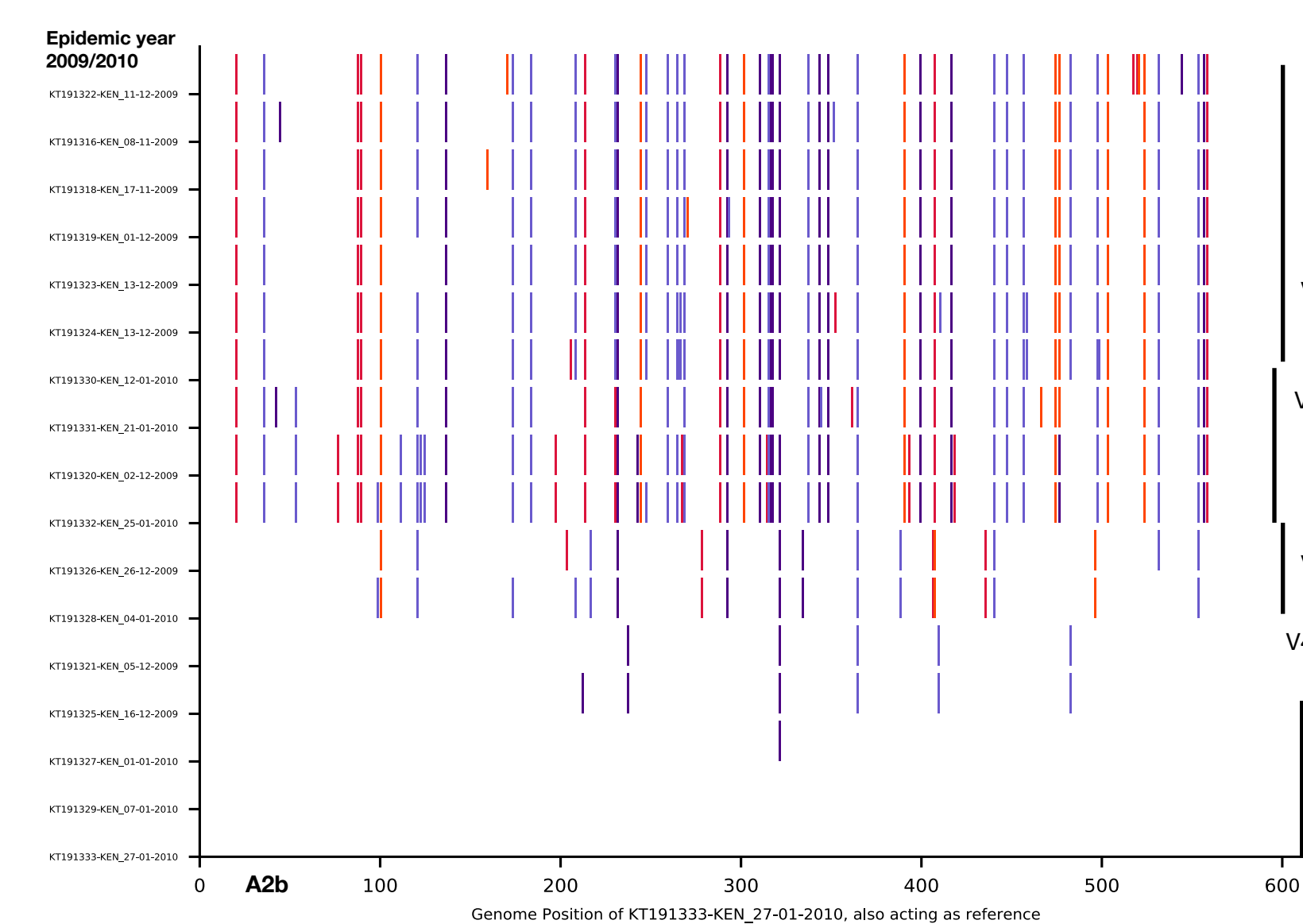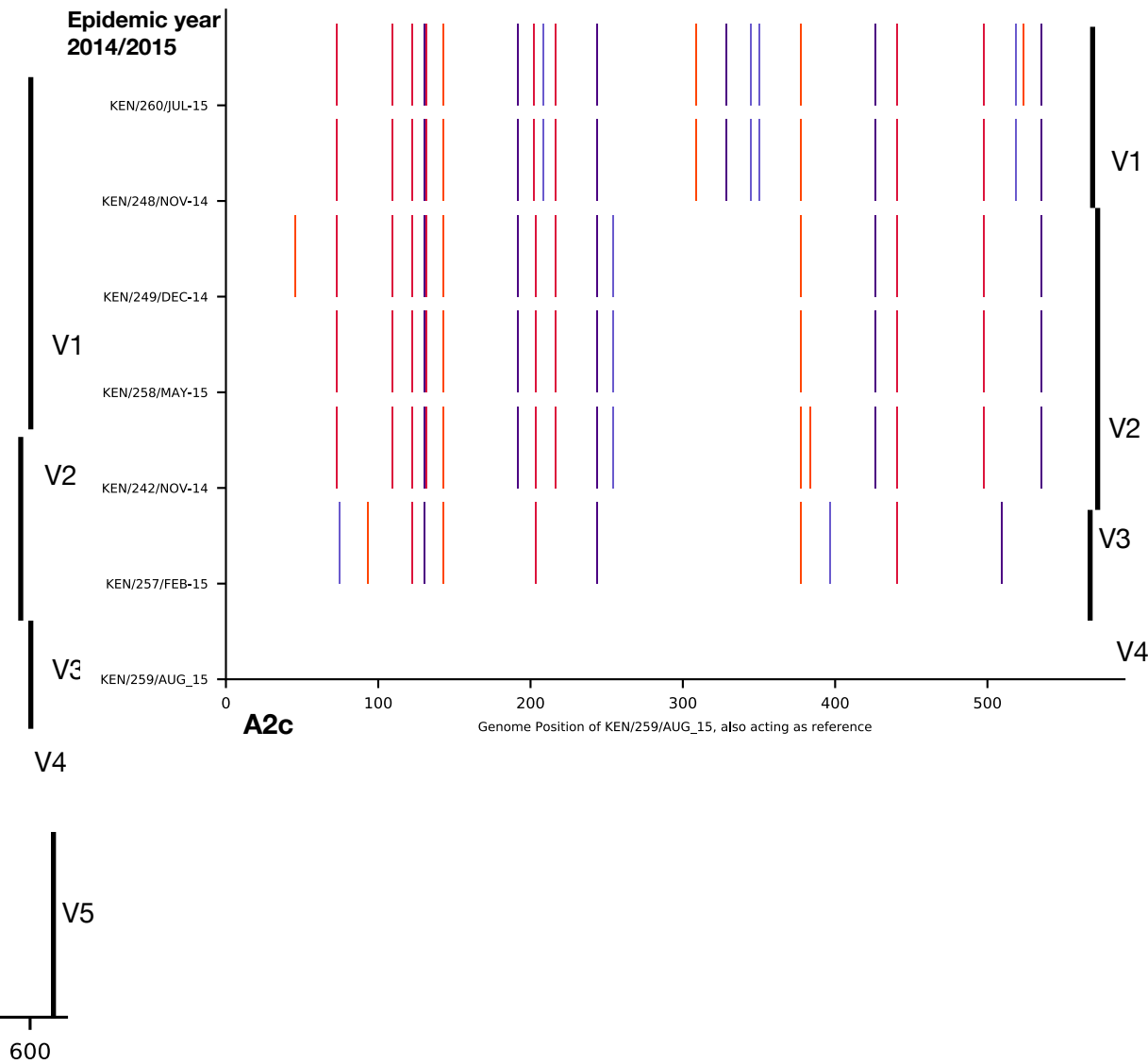

Supplement: Supplementary file 4 — Changes in G gene nucleotide sequences for sequences collected from Kilifi. Sequences were aligned by subgroup and by epidemic year for Clade A2b and A2c. Nucleotide differences were determined and indicated by vertical colored bars. Orange red represent Adenine (A), Crimson represent Thymine (T), Indigo represents Guanine (G) and slate blue represent Cytosine (C) nucleotide. (PDF 261 kb) [file 12879_2019_4381_MOESM4_ESM.pdf]

Epidemic year  
2013/2014

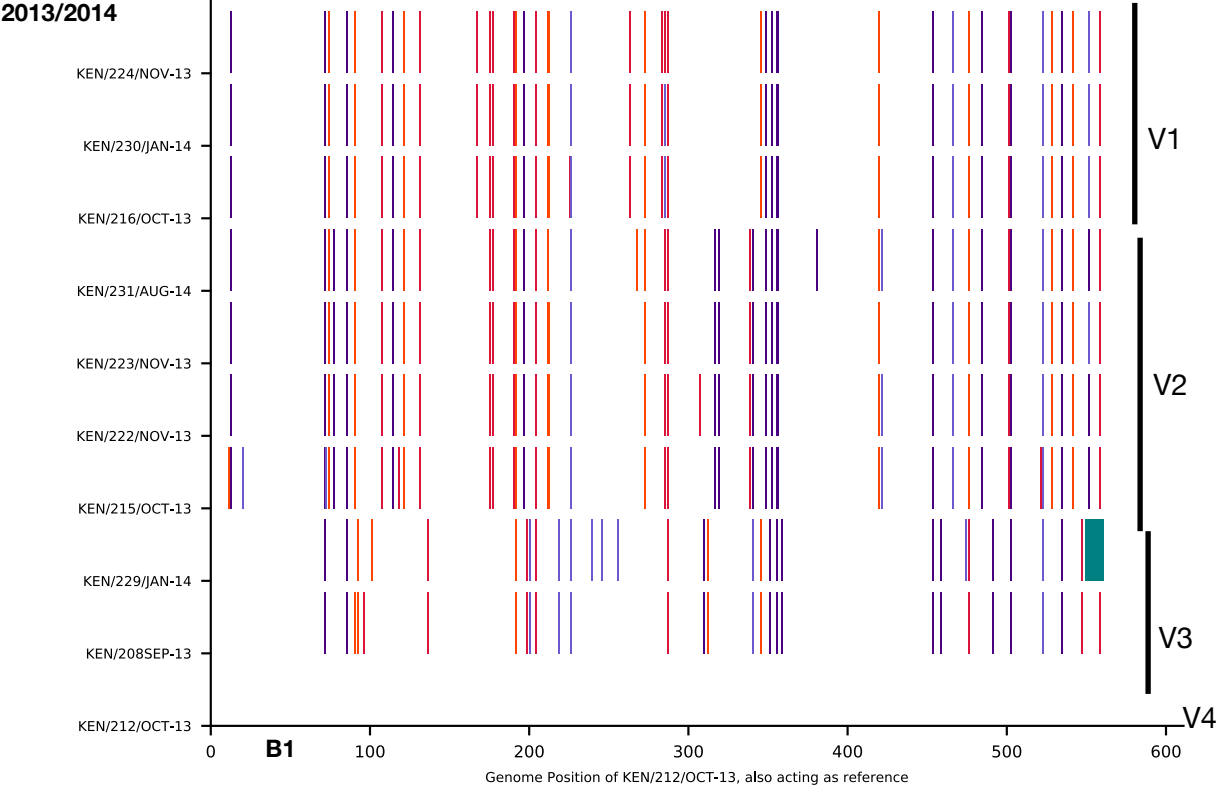

Epidemic year  
2014/2015

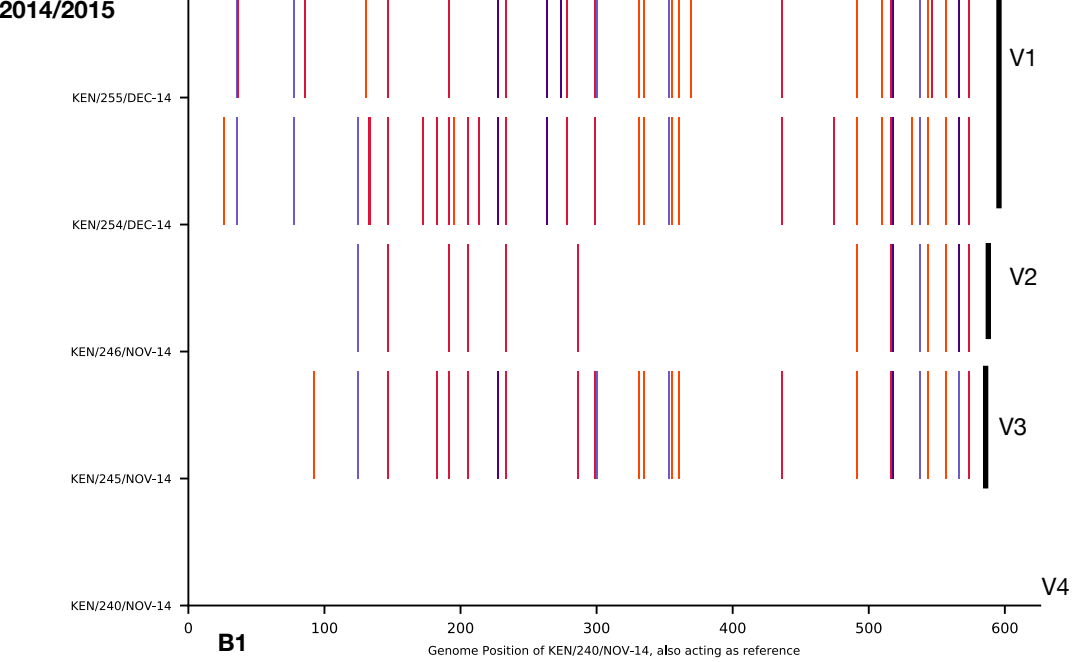

V= variant

Epidemic year  
2011/2012

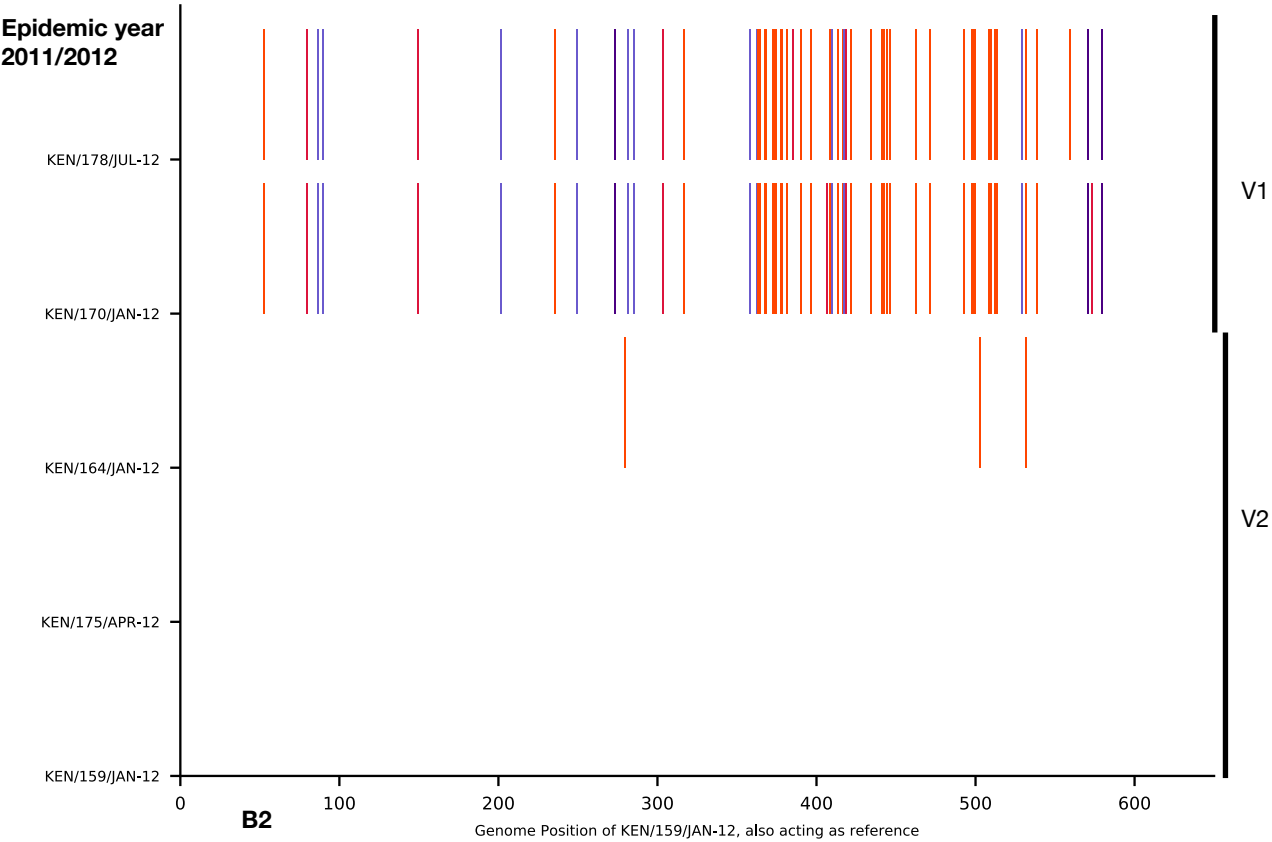

Epidemic year  
2013/2014

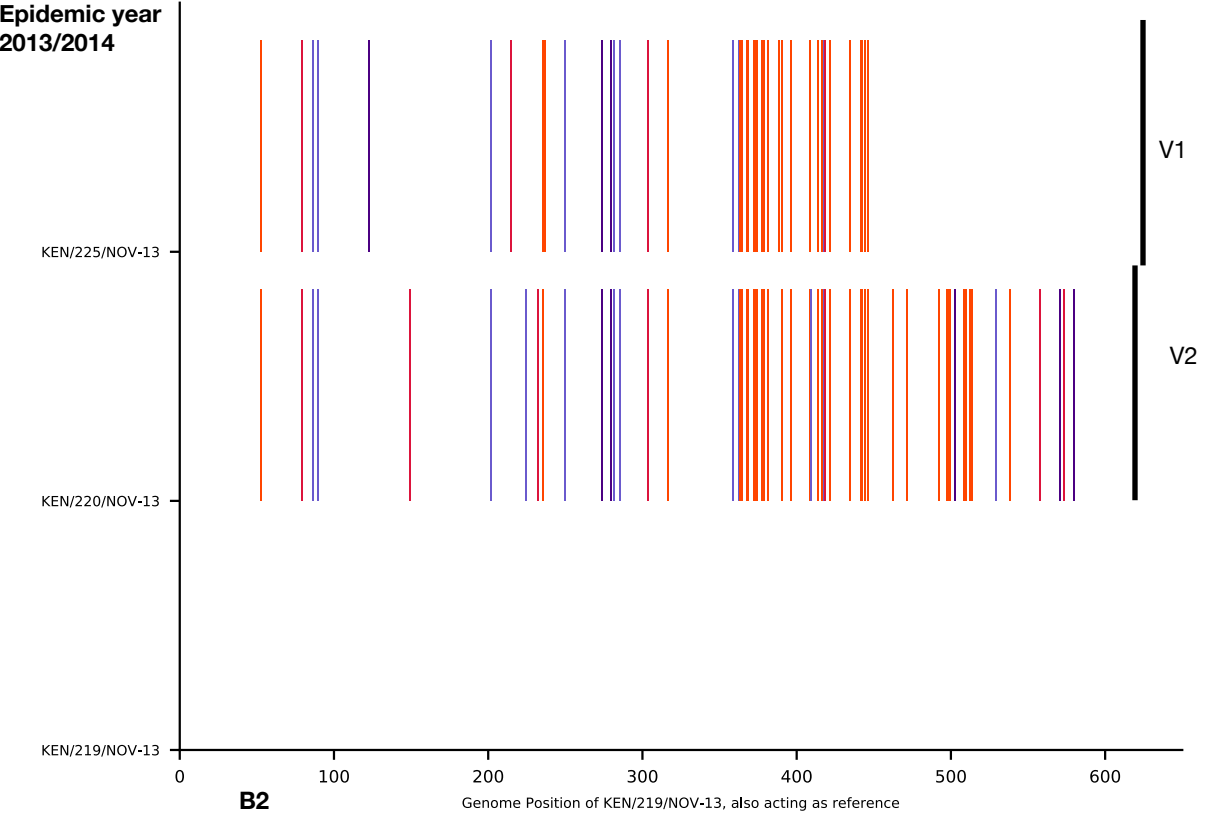

Supplement: Supplementary file 5 — Changes in G gene nucleotide sequences for sequences collected from Kilifi. Sequences were aligned by subgroup and by epidemic year for subgroups B1 and B2. Nucleotide differences were determined and indicated by vertical colored bars. Orange red represent Adenine (A), Crimson represent Thymine (T), Indigo represents Guanine (G) and slate blue represent Cytosine (C) nucleotide. (PDF 167 kb) [file 12879_2019_4381_MOESM5_ESM.pdf]
