# Supplementary material for: Human metapneumovirus prevalence and patterns of subgroup persistence identified through surveillance of pediatric pneumonia hospital admissions in coastal Kenya, 2007–2016
Source: BMC Infect Dis. 2019 Aug 30;19:757. doi: 10.1186/s12879-019-4381-9 (PMC6716807; doi:10.1186/s12879-019-4381-9)
Supplement: Supplementary file 6 — Parameter estimates, dN/dS ratios, positively selected sites and log likelihood scores for F and G gene nucleotide sequences for the HMPV subgroups identified between 2007 and 2016. (PDF 48 kb) [file 12879_2019_4381_MOESM6_ESM.pdf]

S6

Parameter estimates,dN/dS ratios,positively selected sites and log likelihood scores for F and G gene nucleotide sequences for Kilifi HMPV genotypes.							
	Subgroup and Model		dN/dS[a]	Parameter estimates		PSS	loglikelihood
	A2c	M0	0.10386	ω=0.10386		none	-2380.250943
		M1	0.0865	p_0 =0.91,P_1 =0.09 ;{ω_0 =0, ω_1= 1}		Not allowed	-2371.8481
		M2	0.1129	p_0 =0.95,P_1 =0.00, P_2 = 0.05;{ω_0 =0, ω_1= 1, ω_2 = 2.47}		none	-2370.568413
F gene		M3	0.1128	p_0 =0.40,P_1 =0.56, P_2 =0.05 ;{ω_0 =0, ω_1= 0, ω_2 =2.46 }		none	-2370.568435
	A2b	M0	0.1539	ω=0.15394		none	-769.367785
		M1	0.101	p_0 =0.89,P_1 =0.10 ;ω_0 =0, ω_1= 1		Not allowed	-749.78974
		M2	0.2566	p_0 =0.91,P_1 =0.06, P_2 =0.03 ;{ω_0 =0, ω_1= 1, ω_2 = }		none	-744.10291
		M3	0.242	p_0 =0.93,P_1 =0.00, P_2 = 0.07;{ω_0 =0, ω_1= 0, ω_2 = 3.40}		none	-744.551601
	B1	M0	0.0144	ω=0.01443		none	-614.044605
		M1	0.0144	p_0 =1.0,P_1 =0.00 ;ω_0 =0.01, ω_1= 1		Not allowed	-614.044661
		M2	0.0144	p_0 =1.0,P_1 =0.00, P_2 =0.00 ;{ω_0 =0.01, ω_1= 1, ω_2 =9.60}		none	-614.044538
		M3	0.0144	p_0 =0.31,P_1 =0.33, P_2 =0.35 ;ω_0 =0.01,ω_1= 0.01, ω_2 =0.1		none	-614.044538
	B2	M0	0.1198	ω=0.11980		none	-717.782054
		M1	0.1036	p_0 =0.90,P_1 =0.10 ;ω_0 =0, ω_1= 1		Not allowed	-709.823007
		M2	0.1418	p_0 =0.92,P_1 =0.00, P_2 =0.08 ;{ω_0 =0, ω_1= 1, ω_2 =1.83 }		none	-709.170371
		M3	0.1418	p_0 =0.67,P_1 =0.25, P_2 = 0.08;ω_0 =0, ω_1= 0, ω_2 = 1.83		none	-709.170371
	A2c	M0	0.3822	ω= 0.38219		none	-943.348921
		M1	0.4104	p_0 =0.66,P_1 =0.38 ;{ω_0 =0.05, ω_1= 1 }		Not allowed	-941.642281
G gene		M2	0.4652	p_0 =0.94,P_1 =0.00, P_2 =0.06 ;{ω_0 =0.25, ω_1= 1, ω_2 =3.58 }		17T,24S,28N	-941.244453
		M3	0.4652	p_0 =0.07,P_1 =0.87, P_2 = 0.06;ω_0 =0.25, ω_1=0.25, ω_2 =3.58}		none	-941.244453
	A2b	M0	0.56976	ω=0.5698		none	-2032.157168
		M1	0.5014	p_0 =0.63,P_1 =0.36 ;{ω_0 =0.21, ω_1= 1}		Not allowed	-2021.329797
		M2	0.8112	p_0 =0.78,P_1 =0.18, P_2 =0.04 ;{ω_0 =0.3, ω_1= 1, ω_2 =8.16}		87L,123Q,135P	-2006.200586
		M3	0.8052	p_0 =0.25,P_1 =0.71, P_2 =0.05 ;{ω_0 =0.13, ω_1=0.58, ω_2 =7.92 }		none	-2006.045905
	B1	M0	0.7821	ω= 0.78214		none	-1512.591846
		M1	0.6463	p_0 =0.35,P_1 =0.64 ;ω_0 =0, ω_1= 1		Not allowed	-1503.175065
		M2	1.021	p_0 =0.39,P_1 =0.51, P_2 =0.10 ;ω_0 =0.07, ω_1= 1, ω_2 =4.82		96H	-1497.511713
		M3	1.0502	p_0 =0.62,P_1 =0.35, P_2 =0.03 ;ω_0 =0.22, ω_1= 1.85, ω_2 =7.92		none	-1497.306571
	B2	M0	1.20832	ω= 1.20832		none	-1646.028324
		M1	0.8094	p_0 =0.19,P_1 =0.80 ;ω_0 =0, ω_1= 1		Not allowed	-1642.966656
		M2	1.494	p_0 =0.21,P_1 =0.56, P_2 =0.23 ;ω_0 =0.07, ω_1= 1, ω_2 =3.95		12T,127I	-1635.14972
		M3	1.52	p_0 =0.43,P_1 =0.50, P_2 =0.06 ;{ω_0 =0.27, ω_1=1.95, ω_2 =6.87}		none	-1635.013273
